# Editorial: Insights in general pediatrics and pediatric emergency care: 2022

**DOI:** 10.3389/fped.2023.1181390

**Published:** 2023-04-11

**Authors:** Joe Kossowsky, Silvia Bressan, Jérémie F. Cohen

**Affiliations:** ^1^ Department of Anesthesiology, Critical Care and Pain Medicine, Boston Children’s Hospital, Boston, MA, United States; ^2^ Department of Anesthesia, Harvard Medical School, Boston, MA, United States; ^3^ Department of Women’s and Children’s Health, University of Padova, Padova, Italy; ^4^ Department of General Pediatrics and Pediatric Infectious Diseases, APHP, Necker-Enfants Malades Hospital, Université Paris Cité, Paris, France

**Keywords:** choosing wisely, CPR—cardiopulmonary resuscitation, testicular torsion, intranasal fentanyl, domestic accidents, pediatric emergencies

**Editorial on the Research Topic** Insights in general pediatrics and pediatric emergency care: 2022

## Background

Following the success of the 2021 edition, the Research Topic “Insights in General Pediatrics and Pediatric Emergency Care 2022” received interest from authors from diverse geographical regions. This Research Topic includes five articles, selected from eight submitted manuscripts. The papers tackle important challenges for both in-hospital and out-of-hospital care, covering broad themes such as general appropriateness of care in pediatrics, and more selective topics, including nurse-directed administration of intranasal fentanyl in the pediatric emergency department, effectiveness of ventilations during cardiopulmonary resuscitation (CPR), self-efficacy of domestic helpers in pediatric emergency management at home, and predictors of testicular salvage in patients with testicular torsion. Several study designs were employed, including a literature review, retrospective chart analysis, a survey, and a simulation study. These various contributions offer opportunities to reflect upon and improve the prevention, diagnosis, treatment, and prognosis of several common pediatric medical conditions.

## Summary of published studies

In a narrative review, Trapani et al. provided an extensive overview of the Choosing Wisely campaign in pediatrics, an initiative to reduce overdiagnosis and overtreatment in healthcare. It was reported that over 80 organizations from 25 countries have embraced this campaign, and providers in various specialties have begun compiling lists of frequently used tests and treatments that are widely seen as unnecessary. This narrative review provided background on the inception of the Choosing Wisely campaign, discussed the spread of the campaign from the US and Canada to other countries, and highlighted the importance of the campaign's recommendations in pediatrics. Obstacles towards applying the campaign's recommendations to the pediatric population and the necessity of creating implementation plans were both discussed.

In a brief research article, Romano et al. aimed to report on nurse-directed, intranasal fentanyl administration to children presenting with musculoskeletal pain secondary to trauma from 2019 to 2021 at the University Children's Hospital of Bern in Switzerland, using a retrospective design. Of the 314 patients included in this study, two experienced a mild adverse event of vertigo, and one experienced a serious adverse event of syncope and hypoxia, notably when the nurse-directed protocol was violated. These findings highlight that nurse-directed, intranasal fentanyl administration seems a safe and effective option for pediatric acute pain management in the emergency room.

In a manikin simulation study, Geerts et al. aimed to verify the European Resuscitation Council's guidelines of five initial rescue breaths in infants, as opposed to two, in order to deliver two effective ventilations. A convenience sample of 112 medical students from Ghent University, Belgium, were given CPR training using bag-mask ventilation before dividing into pairs to perform five cycles of 2-person CPR on an infant manikin. Tidal volume of the rescue breath was measured using a Resusci Baby QCPR manikin. While about half of the medical student pairs delivered two effective rescue breaths as their initial two breaths, there was a significant and substantial increase (+17%) in the number of student pairs able to deliver at least two effective rescue breaths when delivering the full set of five breaths. The results of the study corroborate the recommendation of the European Resuscitation Council to administer five initial rescue breaths in infants.

In a cross-sectional survey, Ho et al. assessed the self-efficacy of domestic helpers in pediatric emergency management at home. A convenience sample of 385 domestic helpers in Hong Kong completed the Self-Efficacy of First Aid in Unintentional Injury at Home questionnaire, a validated tool assessing the knowledge and self-efficacy of emergency management. It was found that the self-efficacy of domestic helpers in emergency management was low (mean score 29/48 points), and both completeness of first-aid training and educational levels were predictors for domestic helpers' self-efficacy. The findings from the study emphasize the need for adequate first aid and emergency management training for domestic helpers.

Finally, in a retrospective study, Chen et al. investigated the predictive value of hematological parameters in testicular salvage in patients with testicular torsion. Children presenting with testicular torsion (*n* = 167) to Shenzhen Children's Hospital, China, between 2010 and 2021 were included for analysis and compared against healthy children (*n* = 100). Patients with testicular torsion were further characterized into two groups based on their outcomes: successful testicular salvage and failed testicular salvage. It was found that shorter symptom duration, lower degree of spermatic cord torsion, and lower monocyte count were predictors of successful testicular salvage. Additionally, monocyte count in the failed salvage group was found to be significantly higher than in the successful salvage and control groups. This highlights the potential utility of using monocyte count as a preoperative biomarker for the success of testicular salvage.

## Commentary

Once again, the Research Topic “ Insights in General Pediatrics and Pediatric Emergency Care” has contributed to sharing with our broad readership of clinicians and researchers new insights on diverse topics, including clinical challenges we face in different pediatric settings. All the papers' findings have practical implications for the care of children, spanning from prevention to prognosis. Notably, by shedding light on the origin, development, and spread of the Choosing Wisely campaign recommendations, Trapani's review article reminds us that, although “doing more by doing less” is often difficult to accept by clinicians and patients (and their parents), greater efforts should be directed towards effective and sustainable implementation strategies of evidence-based practices, customized to each setting characteristics. The original research articles provide additional knowledge to optimize effective provision of pediatric emergency care, as well as further evidence to support and encourage current practices. Confident that colleagues from around the world will find this content useful, we are now looking forward to welcoming submissions describing the state of the art, outlining recent developments and future perspectives to move the field forward for the next edition of our Research Topic “Insights in General Pediatrics and Pediatric Emergency Care”.

